# Ethnic Residential Segregation: A Multilevel, Multigroup, Multiscale Approach Exemplified by London in 2011

**DOI:** 10.1007/s13524-015-0430-1

**Published:** 2015-10-20

**Authors:** Kelvyn Jones, Ron Johnston, David Manley, Dewi Owen, Chris Charlton

**Affiliations:** School of Geographical Sciences and Centre for Multilevel Modelling, University of Bristol, Bristol, BS8 1SS UK; Centre for Multilevel Modelling, University of Bristol, Bristol, BS8 1SS UK; OTB - Research for the Built Environment, Faculty of Architecture and the Built Environment, Delft University of Technology, PO Box 5030, 2600 GA Delft, The Netherlands

**Keywords:** Segregation, Ethnicity, Multilevel modeling, Multiple scales, London

## Abstract

We develop and apply a multilevel modeling approach that is simultaneously capable of assessing multigroup and multiscale segregation in the presence of substantial stochastic variation that accompanies ethnicity rates based on small absolute counts. Bayesian MCMC estimation of a log-normal Poisson model allows the calculation of the variance estimates of the degree of segregation in a single overall model, and credible intervals are obtained to provide a measure of uncertainty around those estimates. The procedure partitions the variance at different levels and implicitly models the dependency (or autocorrelation) at each spatial scale below the topmost one. Substantively, we apply the model to 2011 census data for London, one of the world’s most ethnically diverse cities. We find that the degree of segregation depends both on scale and group.

## Introduction

The massive research literature on residential segregation in general, and ethnic residential segregation in particular, has widely recognized of the importance of spatial scale to its measurement, reflecting the different scales at which the decision-making processes regarding where to live within a city’s residential fabric are made. Among those able to exercise at least some choice within the housing market—that is, excluding those allocated to a dwelling by a public sector agency—decisions are made regarding the following: (1) the part of a city to live in (e.g., inner city or outer suburb); (2) the sector of a city, taking into account access to places of work, schooling, leisure and cultural activities, and so on; and (3) the particular dwelling within a chosen area, partly reflecting access to both local services plus significant others, such as kin and coethnics. Thus, members of some groups may be substantially concentrated in particular parts of a metropolitan area only but widely scattered within them; others may occupy tight, near-exclusive clusters of dwellings that are distributed across several different segments of the housing market.

Measuring the degree to which groups are spatially segregated invokes (implicitly, at least) the well-known modifiable areal unit problem (MAUP) as applied to spatially aggregated census and other data. Researchers have long recognized that a measured level of segregation—using a range of standard procedures, such as the much-deployed indices of dissimilarity and segregation—is a function of both the scale of aggregation (see Jones and McEvoy 1978; Logan et al. 2015; Manley 2014; Wong 2003; Woods 1976) and the particular set of areas used at any one scale. Scale is important for understanding the causes and impact of segregation, and there is no one correct scale with which to measure it. Consequently, we argue in this article the need for analysis at multiple scales and to do so simultaneously to enable an assessment of the degree of segregation at one scale *net* of another. One of the few studies to do this (Fischer et al. 2004) not only looked at the level of segregation at different scales within the United States (from region down to census tract) but also decomposed those levels to identify the relative importance of each (see also Voas and Williamson 2000; Johnston et al. (2003) and Fowler (2015) both also made the case for a multiscale approach but like those just referred to, did not do so in a modeling framework). The modeling approach adopted here does not resolve the MAUP. Like most other studies, its findings are constrained by the spatial units deployed, most, if not all, of which are pre-given. Use of other spatial architectures may generate different findings, although we believe that the general patterns identified here are unlikely to be contradicted, which is an assumption that can be fully evaluated only with future extensive simulation studies.

The approach also needs to be multigroup because many city populations comprise more than one substantial ethnic minority alongside the dominant, usually majority, group (Reardon and Firebaugh 2002). The investigation of ethnic residential segregation in London undertaken here, for example, has to analyze simultaneously the distributions of 13 major ethnic groups forming a city, according to Sturgis et al. (2014:1291), “. . . with a justifiable claim to be the most ethnically diverse, not just in the UK, but in the world.”

Many studies of segregation published over the last near-century have recognized its multigroup, multiscale nature, but with few taking into account Tranmer and Steel’s (2001:947) argument, demonstrated both theoretically and empirically, that if a model is specified excluding an important level (or spatial scale), “the effects of the levels above the highest level included in the analysis will be reflected in estimated components for the highest level included”: a micro-level segregation measure could be inflated if a macro-level pattern is omitted/ignored. Almost without exception, however, the chosen measures have been descriptive only, without taking into account the natural variation that underpins any distribution of population groups across a series of defined spatial units, especially where small numbers of individuals are a common component of many of those distributions using small spatial units (for overviews of that massive literature, see Reardon and Firebaugh 2002; Reardon and O’Sullivan 2004). Even the technically most-sophisticated essays exploring the multiscale nature of ethnic residential segregation (e.g., Lee et al. 2008; Östh et al. 2014, 2015; Reardon et al. 2008, 2009; Wright et al. 2011) provide descriptive measures only, without any formal modeling that incorporates uncertainty. This article addresses that lacuna, deploying a multilevel modeling strategy for the first time in the analysis of multigroup, multiscale ethnic segregation patterns.

We address three related questions: What is the extent of ethnic residential segregation in London in 2011; which ethnic groups are most segregated; and at which spatial scales are they segregated? These questions are set in an explicit inferential and modeling framework that is essential when dealing with the uncertainty that arises with the analysis of small absolute counts. This article has a threefold structure: we consider the nature of the available fine-grained ethnic census data; we develop the multilevel multiscale, multigroup modeling framework; and we apply it to London.

## Methodology

### The Census Data

The data to be analyzed comprise a set of counts for a very large table for all usual residents defined in terms of 13 main categories of ethnicity, at the finest geographical level of detail that is available for the 2011 Population Census of England and Wales.^1^ Our hierarchy of areas in London comprises four sets of nested spatial units employed in reporting these data. At the largest scale are the 32 London boroughs, which ranged in population in 2011 from 158,649 (Kensington and Chelsea) to 363,378 (Croydon). The City of London, which is not treated as a separate borough here,^2^ functions as a service center rather than a residential district, and has a much smaller population of less than 10,000. The boroughs have formally existed for more than 50 years and are the principal subdivisions of the administrative area of the Greater London County. Each borough is governed by a borough council, which oversees the majority of local government services, such as social services, waste collection, roads, and schools.^3^ The boroughs also reflect the history of the growth of London and can be quite distinctive in character (Manley et al. 2015), with London being described as a city with 33 small “cities” within it.^4^

At the smallest areal scale are the 25,086 output areas (OAs), defined to have a compact shape and to minimize within-area and maximize between-area variation in housing tenure and dwelling type, nested within the boroughs and their electoral wards, and with size constraints. Their mean population was 326, with a standard deviation (SD) of 83. These very small areas were defined by automatic zoning algorithms after the individual and household census data had been collected (Cockings et al. 2011). The OAs were combined in the census output tables —again, using the same criteria to maximize their social homogeneity—into lower-level super output areas (LSOAs), with a mean population of 1,691 (SD = 263); and middle-level super output areas (MSOAs), with a mean population of 8,315 (SD = 1,448). Spatially, the OAs are very fine-grained, having a median radius of 105 meters, with the mean number of OAs in a borough being 759. Typically, there are five OAs in an LSOA, and five LSOAs in a MSOA. The units at each scale nest exactly within each other in a hierarchical fashion.

Within this spatial framework, we analyze the distributions of the 13 largest ethnic groups, as defined by the UK Office of National Statistics and deployed in the 2011 census, using questions that allowed respondents to state their ethnic identity within a constrained set. Those 13 groups comprise a disparate set of categories, defined on separate criteria (e.g., country of ancestry, skin color). They form five separate sets:

1. White population groups, which comprise the following:
  White British: The dominant group (some 49 % of the London total)
  White Irish: Those claiming Irish citizenship and/or ancestry
  White Other: A heterogeneous category comprising those claiming citizenship and/or ancestry of a wide range of countries, including the rest of Europe, much of the British Commonwealth, and the United States of America
2. Black population groups, which comprise the following:
  Black Africans: a heterogeneous group mainly consisting of those with either East or West African ancestry
  Black Caribbeans^5^
3. Asian population groups, comprising those claiming the following ethnic identities:
  Indian
  Pakistani
  Bangladeshi
  Chinese
4. Mixed-ethnicity groups, comprising those claiming the following identities:
  White and Black Caribbean
  White and Black African
  White and Asian
5. Arab population group.

Most studies of segregation select a reference to which the distribution of different groups is then compared. The dominant population is usually chosen as the base—in this case, the White British—and other ethnicities are compared with it. One of the advantages of the model-based methodology that we employ is that we can compare all groups against a theoretically even expected distribution. This is derived from a two-step process. First, the proportion of the total London population in each of the 13 groups is calculated. Then an expected count is derived for each of the 13 cells in each row of the (13 × 25,086) data matrix by multiplying the total population for each OA by these London proportions. If the observed count exceeds the expected, there is a greater number of people locally than would be expected from their citywide distribution. If there is no geographical segregation whatsoever, the expected count would equal the observed count in each and every area and for each and every group, and these relative rates of the observed to the expected will all be 1. If the ratio of the observed to the expected count is less than 1, the group is underrepresented in that area. The extent of the under- and overrepresentation for each group is its segregation and is the subject of the modeling here.

## Model Specification and Estimation

### Why Model?

The aim is to develop an explicit model-based approach in which segregation is summarized by a variance term around a mean. Put simply, if there is no variance beyond chance, each area will have the same underlying ethnic experience. Systematic underlying differences in the relative rates between places are shown by a large variance term, beyond what we could have observed from chance alone. As such, this method builds on the recent analysis of school segregation (Leckie and Goldstein 2015; Leckie et al. 2012) and indeed on the long-forgotten paper of Kish (1954), who examined residential differentiation. The new development here is that whereas Leckie and colleagues and Kish used a binomial model in which the outcome is a proportion, we use a Poisson model in which the outcome is a count of the number of people. The Poisson is highly suitable for low absolute counts because many OAs have small numbers in many of the ethnic categories. Moreover, the Poisson formulation allows the comparison of each and every ethnicity, and no particular ethnicity has to be chosen as the reference category as in the binomial logit model; rather, the comparison is with a theoretically even distribution. Our methodology also builds on the pioneering study by Moellering and Tobler (1972), who decomposed variation for predetermined spatial aggregates but only for continuously measured, ratio-scale data. In contrast, we have rates based on a varying numerator and denominator. The resultant model allows for the simultaneous analysis of multiple ethnic groups at multiple scales in an explicit modeling framework that allows us to put confidence intervals (Bayesian credible intervals) around the estimates. This overall model also allows us to see the extent to which members of each ethnic group co-locate with other groups at each scale.

Although one may presume that a total population—a census—does not require an inferential modeling approach (as strongly insisted by Gorard 2007), this is definitely not the case here. An important characteristic of these fine-grained data is their uneven absolute size. Although the mean count is some 22 people of a particular ethnicity in an OA, it ranges from 0 to 736 with a median of just 5 and a lower quartile of just 1, indicating the extent of the clustering of different ethnic groups, as shown in descriptive analyses of London (Johnston et al. 2014, 2015). The approximate standard error of the log of a relative rate based on an observed to expected ratio is inversely proportional to the square root of the observed count (Breslow and Day 1987: equation 2.9). Consequently, we can anticipate a great deal of stochastic variation and do not want to be misled by this natural variation. Indeed, common descriptive indices such as the *D* Dissimilarity Index are known to suffer from the upward bias of the null—showing systematic segregation when there is none—when small counts are analyzed (Allen et al. 2015; Carrington and Troske 1997).^6^ In contrast, we consider the observed counts as an outcome of a stochastic process that could produce different results under the same circumstances. It is this underlying process, or relative risk, that is of interest, and the actual observed values give only an imprecise estimate of this. We need a method that estimates the differences in the “true” ethnicity rates shorn of random noise.

### A Two-Level Model for OAs

To introduce the model, we begin with a two-level model in which individuals are nested within OAs, and we do so for just two ethnic groups: Black Africans and Black Caribbeans. We will later extend this to multiple scales and multiple groups. This basic model is specified as follows:

$$ \begin{array}{l}{O}_{ij}\sim Poisson\left({\uppi}_{ij}\right)\hfill \\ {}Lo{g}_e\left({\uppi}_{ij}\right)=Lo{g}_e\left({E}_{ij}\right)+{\upbeta}_{1j} Africa{n}_{ij}+{\upbeta}_{2j} Caribbea{n}_{ij}\hfill \\ {}{\upbeta}_{1j}={\upbeta}_1+{u}_{1j}\hfill \\ {}{\upbeta}_{2j}={\upbeta}_2+{u}_{2j}\hfill \\ {}\left[\begin{array}{c}\hfill {u}_{1j}\hfill \\ {}\hfill {u}_{2j}\hfill \end{array}\right]\sim N\left(0,\left[\begin{array}{cc}\hfill {\upsigma}_{u1}^2\hfill & \hfill \hfill \\ {}\hfill {\upsigma}_{12}\hfill & \hfill {\upsigma}_{u2}^2\hfill \end{array}\right]\right)\hfill \\ {}Var\left({O}_{ij}\left|{\uppi}_{ij}\right.\right)={\uppi}_{ij},\hfill \end{array} $$

where **O**_*ij*_ is the long stacked vector of the observed count for individuals *i* in OAs *j*. This vector has two observations: the count of Black Africans and Caribbeans for each and every OA. The other observed variables are the expected counts (*E*_*ij*_) for each ethnic group if their numbers were distributed evenly according to the total population size of the OA. In addition, two separately coded dummy variables (*African*_*ij*_; *Caribbean*_*ij*_) identify which stacked count represents which ethnicity.

The counts are modeled in a Poisson regression model,^7^ where the observed counts are seen as coming from a Poisson distribution with a mean rate of occurrence given by π_*ij*_. However, it is the natural log of the underlying rate that is modeled, and this is achieved by the use of an offset, which is the log of the expected count with a coefficient constrained to 1 (McCullagh and Nelder 1989). Thus, the expected value is effectively treated as a nuisance and allows us to model the underlying relative rate with the response simply being the log of the observed counts rates (difference in logs being equivalent to division in raw values). Moreover, using the log also ensures that we cannot estimate a negative relative rate.

The two intercepts in the model are (1) β_1_, which gives the log average rate across all OAs for Black Africans; and (2) the overall log rate for the Black Caribbean population, β_2_. We anticipate that both of these estimates, when exponentiated, will give the all-London rate for the mean area as 1.^8^ There are allowed-to-vary differences for each OA for each ethnicity (*u*_1*j*_; *u*_2*j*_); a positive value indicates a log rate that is higher than expected given an even distribution or equivalently a relative concentration of that ethnic group. These differences are assumed to come from a joint normal distribution, such that the variance σ_*u*1_^2^ and σ_*u*2_^2^ give the differences between OAs for Black Africans and Caribbeans, respectively. These are our measures of comparative segregation. A useful term is the higher-level OA covariance term, *o*_*u*12_; when standardized by the product of the square root of the variances, this gives the correlation between the differences for the two groups. A negative value indicates that each group is antagonistically located relative to the other; a positive value represents a tendency to co-locate.

At the lowest individual level, the variances are constrained to be equal to the underlying rate for each ethnic group as befits an exact Poisson distribution. Consequently, the model separates the two sources of variation: the variation due to “true” between-OA variation, and that due to stochastic Poisson variability. Equivalently, the lower level of the model is used to model the natural variation of a Poisson variable, whereas the higher level is used to model the extra-Poisson variation of the “true” rates to give a measure of comparative segregation.

The apparent problem, of course, is that we do not have individual data but only aggregate counts for OAs because of confidentiality requirements. However, we can use the device of a pseudo-level in which the OAs are both the *i*s and *j*s in the model. Consequently, there is exactly the same set of units at Level 1 and Level 2, and each Level 2 unit has exactly one Level 1 unit. This views the aggregate counts at Level 2 as consisting of replicated responses for individuals at Level 1. This device allows for extra Poisson variation in the same manner as Browne et al. (2005) achieved for overdispersed binomial multilevel models.

Owen and Jones (2015) discussed a number of ways of turning these variances, which are on a log scale, into a more readily interpretable form. They found that the most appealing is the median rate ratio (MRR) given that this facilitates comparisons between standardized rates. The MRR can be conceptualized as the increased rate (on average; hence, the median) if one compares the rates of two MSOAs chosen at random from the distribution with the estimated variance. If there is no segregation, then the MRR would be 1; a value of 2 would indicate substantial segregation with the randomly chosen area, with the higher rate having twice the rate of the lower area.^9^ The calculation of the MRR is a simple transformation of the variance, and the same operation could be used to derive the 95 % credible intervals (CIs) around each MRR value for significance testing purposes.^10^

The normality assumption of the higher-level differences is obviously a key assumption for the validity of the variance in summarizing the differences in the relative rates. Although inference in multilevel models is typically robust to moderate departures from normality (McCulloch and Neuhaus 2011), severe skewness or outliers can pose problems. Whether these are present can be informally assessed with a normal probability plot. In practice, we have found that the normality assumption is generally met, no doubt due to using the log of the underlying rate. Indeed, in their study of London schools, Leckie and Goldstein (2015) found that parameter estimates obtained through MCMC procedures were not unduly sensitive to the inclusion/exclusion of religiously exclusive outlying schools.^11^

### Multiscale Modeling

The model can readily be extended to work at more scales, and this is the specification for two ethnicities and the three scales of the individual, the OA, and the MSOA that form a strict three-level hierarchy.

$$ \begin{array}{l}{O}_{ijk}\sim Poisson\left({\pi}_{ijk}\right)\hfill \\ {}Lo{g}_e\left({\uppi}_{ijk}\right)=Lo{g}_e\left({E}_{ijk}\right)+{\upbeta}_{1jk} Africa{n}_{ijk}+{\upbeta}_{2jk} Caribbea{n}_{ijk}\hfill \\ {}{\upbeta}_{1jk}={\upbeta}_1+{v}_{1k}+{u}_{1jk}\hfill \\ {}{\upbeta}_{2jk}={\upbeta}_2+{v}_{2k}+{u}_{2jk}\hfill \\ {}\left[\begin{array}{c}\hfill {v}_{1k}\hfill \\ {}\hfill {v}_{2k}\hfill \end{array}\right]\sim N\left(0,\left[\begin{array}{cc}\hfill {\upsigma}_{v1}^2\hfill & \hfill \hfill \\ {}\hfill {\upsigma}_{v12}\hfill & \hfill {\upsigma}_{v2}^2\hfill \end{array}\right]\right)\hfill \\ {}\left[\begin{array}{c}\hfill {u}_{1jk}\hfill \\ {}\hfill {u}_{2jk}\hfill \end{array}\right]\sim N\left(0,\left[\begin{array}{cc}\hfill {\upsigma}_{u1}^2\hfill & \hfill \hfill \\ {}\hfill {\upsigma}_{12}\hfill & \hfill {\upsigma}_{u2}^2\hfill \end{array}\right]\right)\hfill \\ {}Var\left({O}_{ijk}\left|{\uppi}_{ijk}\right.\right)={\uppi}_{ijk},\hfill \end{array} $$

where *O*_*ijk*_ is the long stacked vector of the observed counts for both Black Africans and Caribbeans in cell (type of person) *i* for OA *j* in MSOA *k*. Of the two intercepts, β_1_ gives the log average rate across all MSOAs and OAs for Africans, and the London-wide log rate for Caribbeans is β_2_. There are ethnic-specific differences at the MSOA level (*v*_1*k*_; *v*_2*k*_) and for OAs within MSOAs (*u*_1*k*_; *v*_1*k*_). These differences at each of the higher levels are assumed to come from a joint normal distribution; thus, σ_*v*1_^2^ gives the segregation for Africans at the MSOA level, and we can test whether this is different from the variance for Caribbeans, σ_*v*2_^2^. The higher-level covariance term, when standardized, will give the correlation between the differences at that level between each pair of ethnic groups—that is, the extent to which ethnic groups co-locate at that scale.

### Spatial Modeling of Segregation

Classic measures of segregation (like *D*) are aspatial and depend only on the numerical values in each observation unit (e.g., OAs in London), taking no account of the situation in surrounding areas or the spatial patterning in the rates. Swapping the units spatially so that all the areas with large Caribbean populations are contiguous would produce no change in such an index but may imply much greater segregation. Researchers in recent years have shown considerable interest in developing spatially sensitive measures from two broad viewpoints. From the spatial econometrics perspective (Paelinck and Klaassen 1979), Wong (1998) developed a family of local spatial segregation indices that take account of neighborhood joins, thus taking into account the population characteristics of a wider area (defined *a priori* as in touching boundaries). Wong (2003) extended these measures to work at multiple scales, but those measures were not set in an inferential framework and were based on the observed (and therefore potentially unreliable) local rates. From the spatial smoothing perspective (Fotheringham et al. 2002), Reardon and his coauthors (2004, 2009) also developed spatially sensitive measures. They used a spatially weighted version of the information theory index where the weights are determined *a priori* by some function of the spatial distance between areas. Lee et al. (2008; see also Östh et al. 2014, 2015) used this approach to analyze multiple scales by defining circles of different radii and moving these around the map. They provided an analysis at scales from a 500 meter radius (a pedestrian-based neighborhood) to a 4,000 meter radius (which they call a “macro-local environment”) of nearly 20 square miles.^12^ Their determination of the degree of segregation involved no modeling or inferential framework to deal with unreliable rates resulting from small counts.

Both sets of approaches take into account local spatial autocorrelation or dependency. It may be thought that multilevel models of the type that we are developing here are aspatial, and Elffers (2003) has argued that the between-area variance is invariant to the spatial arrangement of areas. This is undoubtedly true of the standard two-level model, but it is not true in general, for two reasons. First, it is possible to include spatial weights in a multilevel model for the higher areal levels (see Jones and Subramanian 2014b) and thereby estimate both unstructured and spatially structured segregation. Such explicitly spatial multilevel modeling is undergoing rapid development.^13^ Second, the hierarchical model with more than two levels has an implicit spatial dependence, and we now consider this in more detail.

**Fig. 1:**
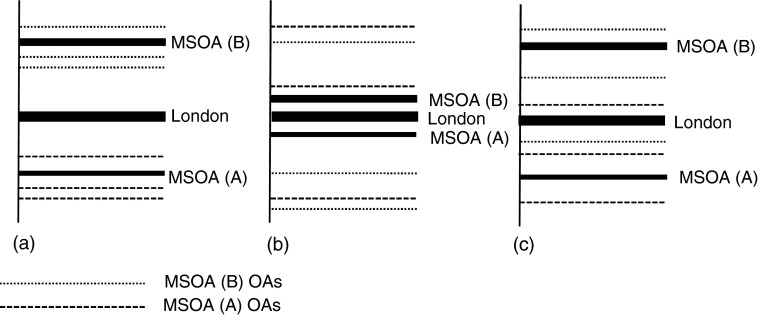
Higher-level variations: (a) large between-MSOA and small between-OA-within-MSOA, (b) small between-MSOA and large between-OAs-within-MSOAs, and (c) substantial between-MSOA and between-OAs-within-MSOA

We begin by stressing that multilevel models analyze the within- and between-differences (Bell and Jones 2015), and the variance σ_*u*1_^2^ in the second formulation no longer summarizes differences between OAs as in the original model but now represents the differences between OAs after taking account of the differences between MSOAs in which they are located (Subramanian et al. 2001). To illustrate the procedure, Fig. 1 uses two higher-scale examples. In each of the three diagrams, the solid line shows the relevant London-wide relative rate of 1—the theoretical even distribution of the population who are, say, Bangladeshi. The city is split at the larger spatial scale into two MSOAs, A and B, each of which is divided at the smaller scale into three OAs. In panel (a) A and B differ substantially in their Bangladeshi relative rates but differ little across their constituent OAs within each MSOA. Segregation is substantial at the larger scale but, holding its extent at that level constant, insubstantial at the smaller scale. Bangladeshis are concentrated in B; but in both A and B, there is little within-MSOA, between-OA variation. Panel (b), on the other hand, shows little difference between MSOA A and B but substantial variation within each; and panel (c) shows substantial variation at both scales. In panel (a), therefore, segregation displays macro-scale variability only; in panel (b), it displays only micro-scale variability; and in panel (c), there is substantial segregation at both scales. Because the multilevel approach measures segregation at one scale net of the others, it does not inevitably mean that the finer scale is necessarily the most segregated.

The key notion in the model is that the highest-level difference is a random, allowed-to-vary departure from a general relationship, and each level’s residual is an allowed-to-vary random departure from the higher-level departure. Consequently, we can calculate a variance partitioning coefficient (VPC: Goldstein 2011; Jones and Subramanian 2014a), which decomposes the total variance into the multiple scales. Moreover, this VPC gives the proportion of the variance between—or the degree of similarity or correlation within—scales, equivalent to the well-known intraclass correlation coefficient (Kish 1954).

Conceptually, in the three-level example, σ_*v*1_^2^ is the between-MSOA variance for ethnic group 1, which is our measure of segregation at this level; σ_*u*1_^2^ is the within-MSOA, between-OA variance for ethnic group 1, which is a measure of segregation at the OA level net of differences at the MSOA level; and σ_*v*1_^2^ + σ_*u*1_^2^ is the between-OA variance for ethnic group 1, which is equivalent to the measure of variance for that scale in the initial two-level model.

Consequently, the proportion of the total variance due to differences between MSOAs, the intra-MSOA correlation is given by

$$ \frac{\upsigma_{v1}^2}{\upsigma_{v1}^2+{\upsigma}_{u1}^2+{\upsigma}_{e1}^2}, $$

where σ_*e*1_^2^ is the within-MSOA, within-OA, between-people variance for group 1.^14^

The proportion of the variance due to differences between OAs, the intra-OA correlation is given by

$$ \frac{\upsigma_{v1}^2+{\upsigma}_{u1}^2}{\upsigma_{v1}^2+{\upsigma}_{u1}^2+{\upsigma}_{e1}^2}. $$

Finally, we can calculate the similarity of OAs within the same MSOAs:

$$ \frac{\upsigma_{v1}^2}{\upsigma_{v1}^2+{\upsigma}_{u1}^2}. $$

**Fig. 2:**
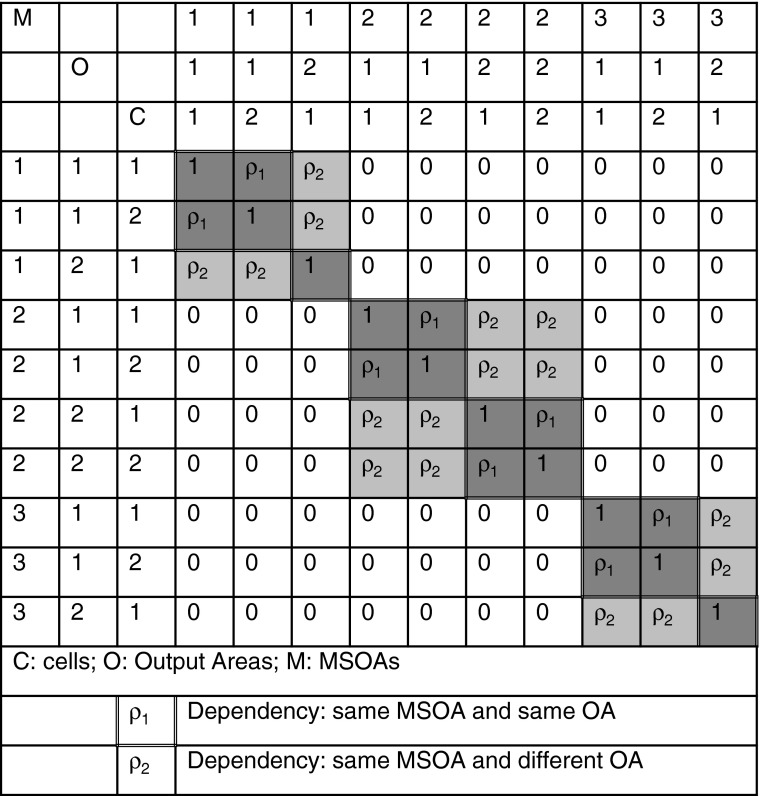
An extract of the dependency structure of a three-level hierarchical model

The hierarchical structure is therefore defining the local neighborhood structure, and we are implicitly modeling spatial dependence. The degree of segregation is not invariant to swapping because we are specifying that a set of OAs belongs within—is hierarchically nested in—a specific MSOA. The inherently spatial nature of this dependence is shown in Fig. 2. Cells (C) are sorted so that they are nested in OAs (O) and MSOAs (M), and it can then be seen that intra-OA correlation (ρ_1_) assesses the degree of correlation in the same MSOA and same OA, while the intra-MSOA correlation (ρ_2_) gives the correlation for those in the same MSOA but different OAs.^15^

The final elaboration of the model is to extend it to more than two ethnicities and to more than three scales. This is trivial in terms of specification but increases estimation time substantially.

### Model Estimation

In the Poisson model, quasi-likelihood empirical Bayes (EB) procedures have been found to overestimate the higher-level variance (Jones and Subramanian 2014b). Consequently, we use full Bayes (FB) procedures for all the models in this article specified with minimally informative prior distributions.^16^ The FB approach allows the calculation of (potentially asymmetric) CIs, which indicate the degree of support enjoyed by different values of our segregation measures, which are estimated without having to rely on asymptotic normality assumptions that are unlikely to hold in applications with a relatively small number of units (as in the case of the Boroughs) and for variance terms that cannot be less than 0.

An important by-product of the MCMC estimation is the deviance information criterion (DIC; Spiegelhalter et al. 2002), which yields an estimate of the badness of fit of the model penalized by model complexity, which in turn is estimated by the degrees of freedom consumed in the fit (pD). A difference in DIC of 10 between two models implies very little support for the model with the higher value of DIC.

We estimate the models with the *MLwiN 2.31* software (Rasbash et al. 2014). The estimates are based on an initial quasi-likelihood estimation, a discarded burn-in of 50,000 simulations to get away from potentially biased results, and a further 100,000 monitoring simulations according to Draper’s (2008) good-practice recommendations. We find it beneficial to use hierarchical centering to obtain less-correlated chains—that is, more informative chains (Browne 2012). The trace of the estimates is evaluated for convergence (shown by lack of trend), and the models were run so that the effective sample size (ESS) of the estimates for each parameter was at least equivalent to 500 independent estimates to characterize the degree of support for parameter values. The estimation takes several days on a standard desktop PC.

## Ethnic Residential Segregation in London, 2011

The motivation for developing the modeling process detailed earlier is to understand segregation in London better for multiple ethnic groups and at multiple scales. To do this, we fit the model for the 13 ethnicities as a sequence: first as cells within OAs, and then additionally adding the intermediate (LSOA and MSOA) to the largest (Borough) scales. Goodness-of-fit is estimated using the DIC criterion, and the improvement at each step is assessed by the change in that measure (ΔDIC). Any change of more than –10 is considered substantial, and Table 1 shows that each additional scale contributes substantially to appreciating the spatial variation in the distribution of all 13 ethnic groups.

**Table 1:** Model goodness-of-fit

Scale	DIC	ΔDIC
OA	1,635,526	––
+LSOA	1,625,241	–10,285
+LSOA & MSOA	1,623,016	–2,225
+LSOA & MSOA & Borough	1,622,704	–312

The key indicator of the modeled degree of segregation for each group at each level is the variance, and the 52 values (13 ethnic groups at four scales) are given in Table 2, along with their 2.5 % and 97.5 % CIs. These can be interpreted in three major ways: (1) within ethnic group, between scales; (2) within scale, between ethnic groups; and (3) cross-group correlations.

**Table 2:** Modeled variances by ethnic group and scale

Ethnic Group/Scale	V	2.5 % CI	97.5 % CI	Ethnic Group/Scale	V	2.5 % CI	97.5 % CI
White British				Indian			
Borough	0.306	0.132	1.030	Borough	1.179	0.705	1.905
MSOA	0.198	0.111	0.743	MSOA	0.364	0.326	0.407
LSOA	0.026	0.025	0.028	LSOA	0.088	0.080	0.096
OA	0.028	0.028	0.029	OA	0.294	0.285	0.304
White Irish				Pakistani			
Borough	0.293	0.174	0.498	Borough	1.827	1.108	2.972
MSOA	0.101	0.091	0.113	MSOA	0.495	0.441	0.566
LSOA	0.031	0.026	0.036	LSOA	0.125	0.109	0.141
OA	0.158	0.152	0.165	OA	0.753	0.727	0.781
White Other				Bangladeshi			
Borough	0.436	0.267	0.709	Borough	3.443	2.025	5.835
MSOA	0.099	0.090	0.110	MSOA	0.627	0.525	0.950
LSOA	0.028	0.026	0.031	LSOA	0.250	0.220	0.284
OA	0.085	0.083	0.088	OA	1.566	1.508	1.626
White and Black Caribbean				Chinese			
Borough	0.294	0.174	0.483	Borough	0.288	0.172	0.483
MSOA	0.188	0.167	0.216	MSOA	0.246	0.218	0.275
LSOA	0.074	0.065	0.084	LSOA	0.107	0.095	0.121
OA	0.338	0.325	0.351	OA	0.588	0.568	0.610
White and Black African				Black African			
Borough	0.263	0.156	0.441	Borough	0.664	0.403	1.101
MSOA	0.187	0.163	0.216	MSOA	0.512	0.453	0.611
LSOA	0.076	0.065	0.088	LSOA	0.147	0.136	0.158
OA	0.551	0.528	0.574	OA	0.354	0.344	0.363
White and Asian				Black Caribbean			
Borough	0.142	0.087	0.233	Borough	1.011	0.595	1.685
MSOA	0.039	0.033	0.046	MSOA	0.402	0.358	0.475
LSOA	0.031	0.025	0.037	LSOA	0.098	0.090	0.106
OA	0.291	0.279	0.303	OA	0.246	0.238	0.254
				Arab			
				Borough	1.423	0.872	2.323
				MSOA	0.285	0.250	0.327
				LSOA	0.138	0.120	0.160
				OA	0.770	0.740	0.799

*Notes:* V = modeled variance; CI = credible intervals.

### Within Ethnic Group: Between Scales

The first set of interpretations looks at each ethnic group separately, exploring differences in the level of segregation across the four scales and establishing whether these differences are statistically substantial using the CIs, as commended in what Cummings (2014) termed the “new statistics,” which abjures the term *statistically significant*. Thus, the first block shows that the White British are more segregated at the Borough than at the MSOA scale, but the overlap between the CIs for the two measures suggests no substantial difference. Those levels of segregation are, however, much larger than those at the smaller two scales, and substantially so. White British people are substantially segregated into particular boroughs and MSOAs within London; within each of those units, however, small-scale variation around the local average is minimal. In general, therefore, each London Borough is relatively homogeneous across its smaller-scale areas in the White British share of the local population: whatever the percentage White British overall (and in most cases, it was either high or low), there is little variation around that figure across its constituent neighborhoods.

To aid comparison, we reexpress the variances as MRRs (Table 3). For interpretation, we can classify these ratios according to well-known effect sizes, as Cohen (1988) recommended originally for odds ratios. Accordingly, values greater than 4.3 indicate very large ratios: MRRs between 2.5 and 4.3 and between 1.5 and 2.5 are considered medium and small, respectively; and MRRs less than 1.5 are treated as low.^17^ The pattern is very clear, as summarized in Table 4: the overwhelming number of MRRs have either low (below 1.5) or small (1.5 to 2.5) values. The only exceptions are (1) five Borough rates, four of which are medium (for Indian, Pakistani, Black Caribbean, and Arab), one large (for Bangladeshi); and (2) one OA rate that is medium (again, for Bangladeshi).

**Table 3:** Modeled variances by ethnic group and scale

Ethnic Group/Scale	MRR	2.5 % CI	97.5 % CI	Ethnic Group/Scale	MRR	2.5 % CI	97.5 % CI
White British				Indian			
Borough	1.695	1.414	2.633	Borough	2.817	2.228	3.731
MSOA	1.529	1.374	2.278	MSOA	1.778	1.724	1.838
LSOA	1.166	1.163	1.173	LSOA	1.327	1.310	1.344
OA	1.173	1.173	1.176	OA	1.677	1.664	1.692
White Irish				Pakistani			
Borough	1.676	1.489	1.960	Borough	3.630	2.729	5.178
MSOA	1.354	1.333	1.378	MSOA	1.956	1.884	2.050
LSOA	1.183	1.166	1.198	LSOA	1.401	1.370	1.431
OA	1.461	1.450	1.473	OA	2.288	2.255	2.323
White Other				Bangladeshi			
Borough	1.877	1.637	2.233	Borough	5.871	3.886	10.016
MSOA	1.350	1.331	1.372	MSOA	2.128	1.996	2.534
LSOA	1.173	1.166	1.183	LSOA	1.611	1.564	1.663
OA	1.321	1.316	1.327	OA	3.299	3.226	3.375
White and Black Caribbean				Chinese			
Borough	1.677	1.489	1.940	Borough	1.668	1.485	1.940
MSOA	1.512	1.477	1.558	MSOA	1.605	1.561	1.649
LSOA	1.296	1.275	1.318	LSOA	1.366	1.342	1.393
OA	1.741	1.723	1.760	OA	2.078	2.052	2.106
White and Black African				Black African			
Borough	1.631	1.458	1.884	Borough	2.176	1.832	2.721
MSOA	1.511	1.470	1.558	MSOA	1.979	1.900	2.108
LSOA	1.301	1.275	1.327	LSOA	1.442	1.422	1.461
OA	2.030	2.000	2.060	OA	1.764	1.750	1.777
White and Asian				Black Caribbean			
Borough	1.433	1.325	1.585	Borough	2.609	2.087	3.449
MSOA	1.207	1.189	1.227	MSOA	1.831	1.770	1.930
LSOA	1.183	1.163	1.201	LSOA	1.348	1.331	1.364
OA	1.673	1.655	1.691	OA	1.605	1.593	1.617
				Arab			
				Borough	3.120	2.437	4.280
				MSOA	1.664	1.611	1.725
				LSOA	1.425	1.392	1.465
				OA	2.310	2.272	2.346

*Notes:* MRR = modeled median rate ratios; CI = credible intervals.

**Table 4:** Summary of the size of the MRR values in Table 3 at the four spatial scales

	MRR			
Scale	Low <1.5	Small 1.5–2.0	Medium 2.0–4.2	Large 4.2<
Borough	1	7	4	1
MSOA	1	7	4	1
LSOA	12	1	0	0
OA	3	9	1	0

The across-scale differences for each of the ethnic groups are shown in Table 3. For 8 of the 13 groups (White Irish, White Other, Indian, Pakistani, Bangladeshi, Black African, Black Caribbean, and Arab), segregation is highest at the Borough scale and second highest at the OA scale, with much lower levels at the MSOA and LSOA scales. Overlapping CIs indicate that those differences are not statistically substantial in several cases; however, the CIs for Borough and OA scales overlap for Pakistanis, but not for Indians and Bangladeshis.

No other group has a pattern similar to the White British: that of continued declining segregation with decreasing scale. The three mixed-ethnicity groups—like the eight identified previously—have their highest segregation levels at the Borough and OA scales, too, but larger for the latter than the former (although in the case of the White-Black Caribbean mixed group, the CIs overlap). The Chinese stand out with much greater modeled segregation at the OA level than any of the other three; within each Borough, a small number of neighborhoods have relatively numerous Chinese residents, but they are not substantially concentrated in particular Boroughs.

In general, therefore, these modeled variances suggest that for most of London’s ethnic groups in 2011, segregation was both a macro- and a micro-scale phenomenon (the Borough and OA, respectively) but not also at the meso-scale (MSOA and LSOA). Most migrant groups are concentrated into particular boroughs, and within them there is significant small-scale local variation given that they are clustered in some parts of the boroughs but not others. The White British are the main exception to this: they, too, are concentrated in particular boroughs, within which there is no local spatial variation. They are represented by panel (a) in Fig. 1, whereas most of the others fall in type (c) (also shown in Fig. 1), and the Chinese are the main exception as type (b) (Fig. 1). The majority White British are concentrated in large blocks of territory, represented here at the Borough scale, in which the subdivisions are homogeneously White, whereas the minority ethnic groups are also concentrated in (a smaller number of) certain boroughs and additionally in certain small blocks within those boroughs.

### Within Scale: Between Ethnic Groups

In these comparisons, the segregation levels (variances) are rank ordered to identify which are the most- and least-segregated ethnic groups at each scale (Table 5). Although there are differences in detail, the general pattern is very clear: the most-segregated groups at all four scales are those with self-assessed Asian (especially South Asian) and Black ethnicities, whereas the least segregated at every scale, too—are the White British, Irish, and Others. The levels of segregation for those claiming a mixed ethnic identity tend to be less than those for the nonwhite group that they partially identify with, but more than for the white populations.

**Table 5:** Rank-orderings of modeled variances for each ethnic group, by scale

Borough	Segregation Level (variance)	MSOA	Segregation Level (variance)	LSOA	Segregation Level (variance)	OA	Segregation Level (variance)
Bangladeshi	3.443	Bangladeshi	0.627	Bangladeshi	0.250	Bangladeshi	1.566
Pakistani	1.827	BAfrican	0.512	BAfrican	0.147	Arab	0.770
Arab	1.423	Pakistani	0.495	Arab	0.138	Pakistani	0.753
Indian	1.179	BCaribbean	0.402	Pakistani	0.125	Chinese	0.588
BCaribbean	1.011	Indian	0.364	Chinese	0.107	White–BA	0.551
BAfrican	0.664	Arab	0.285	BCaribbean	0.098	BAfrican	0.354
White Other	0.436	Chinese	0.246	Indian	0.088	White–BC	0.338
White–BC	0.294	White–BC	0.188	White–BA	0.076	Indian	0.294
Chinese	0.288	White–BA	0.187	White–BC	0.074	White–Asian	0.291
White–BA	0.263	White British	0.124	White Irish	0.031	BCaribbean	0.246
White Irish	0.241	White Irish	0.101	White–Asian	0.031	White Irish	0.158
White British	0.186	White Other	0.099	White Other	0.028	White Other	0.085
White–Asian	0.142	White–Asian	0.039	White British	0.026	White British	0.029

*Note:* BAfrican = Black African; BCaribbean = Black Caribbean; White–BA = Mixed White, Black African; White–BC = Mixed White, Black Caribbean.

Although the 13 groups can be arranged along continua as in Table 5, the differences between adjacent groups are rarely significantly different, especially at the larger spatial scales. At the Borough scale, for example, the CIs overlap between every adjacent pair, but there are differences between nonadjacent pairs.^18^ The first substantial difference (denoted by the underline) along the continuum is between the Bangladeshis (variance (V) = 3.443; CI = 2.025, 5.835) and the Indians (V = 1.179; CI = 0.705, 1.905). The next substantial difference is between Indians and the mixed White–Black Caribbean group (V = 0.294; CI = 0.174, 0.483). No group below that on the continuum differs substantially from the White–Black Caribbeans, suggesting that the 13 groups can be divided into three according to their segregation level at that scale, with the boundaries between the three groups shown by lines in the ranking: (1) three of the four South Asian groups (the most segregated at that scale); (2) the Indians, the two Black groups, and the White Others (less segregated); and (3) the remaining groups, comprising the White British and Irish, the Chinese, and the three mixed groups (the least segregated).

Similar splits are reported in the other three columns of Table 5 for the smaller scales. They show greater variety: more clusters of ethnic groups that differ substantially from their neighbors in their degree of segregation, although segregation levels are generally low at the MSOA and LSOA scales. The greatest degree of substantial variation is at the smallest scale (the OA), which is divided into nine segments in each of which the top-ranked ethnic group has a significantly smaller level of segregation than that at the bottom of the segment above it and where each of the four least segregated groups has a significantly smaller modeled level of segregation from that immediately above it on the continuum. The Bangladeshis are the most segregated at all four scales, and the Arabs and Pakistanis are also highly segregated across the four; at the other extreme, the White British are either the least- or the second least–segregated group (again, as with all of the other comparisons, with segregation at the higher scales held constant).

These findings are in line with those of other descriptive, single-scale analyses of ethnic segregation in London (e.g., Johnston et al. 2015), but also extend them. There is no established theory suggesting which groups should be most or least segregated, let alone of any variations across scales. In general, however, the more recent arrivals are expected to be more segregated than the longer-established groups; those culturally more distinct from the host society are expected be more segregated than those that are less so (most Black Caribbeans are Christians, for example); and those claiming mixed ethnicities are expected to be less segregated than the minority group with which they partially identify but more segregated than the dominant White groups (their mixed identity being an indicator of cultural, and possibly economic, assimilation). The findings reported in Tables 2, 3, and 5 sustain that interpretation. Thus, the predominantly Muslim Bangladeshis and Pakistanis are among the most segregated groups at every scale, for example, but the more heterogeneous other South Asian group (Indians, comprising Muslims and Sikhs as well as the majority Hindus^19^) is less so; and the White and mixed groups are among the least segregated.

In addition, however, the decomposition provided by the modeled variances—the assessed segregation level at each scale is net of that identified at the higher scales—provides information not available from other studies. This is exemplified in two particular cases. The Black Caribbeans are long-established in London—large-scale immigration having been initiated in the late 1940s—and the group has not grown over the most recent decade with few new arrivals (Jivraj and Simpson 2015). They are relatively highly segregated at the macro-scale (i.e., Borough), reflecting the parts of London in which they initially settled, but much less so at the micro-scale (i.e., OA), almost certainly indicative of economic and social mobility over the last few decades; while concentrated in particular parts of London (indicative of inertia in residential decision-making at the macro-scale), they are not strongly clustered within particular smaller areas there—a patterning that distinguishes them from several other more recent and still-expanding (Johnston et al. 2013) immigrant groups, including the Black Africans.

By way of contrast, the relative size of the segregation measures for the Chinese is the inverse of that for the Black Caribbeans. They rank ninth among the 13 groups for their degree of segregation at the Borough scale, for example, but fourth at the OA scale (and the CIs in Table 3 indicate a statistically significant difference between the two). Across London as a whole, therefore, the Chinese are relatively widely distributed—certainly more so than the other Asian ethnic groups. (The Chinese MRR at that scale is significantly smaller than those for the Bangladeshis, Pakistanis, and Indians: see Table 2.) Within those areas where they are relatively concentrated, however, they are more clustered at the most local scale (at the OA level) than all but three other groups, including Indians. As with Black Caribbeans, therefore, these multiscale estimates net of any segregation at higher scales not only confirm our general appreciation of which groups are more or less segregated; they also add an important indication of the statistical significance of those differences and demonstrate that relative levels of segregation vary by scale, indicative, among other factors, of the groups’ length of settlement in the city and its degree of economic and cultural assimilation into the wider society.

### Cross-Group Correlations

This final analysis explores the degree to which the various ethnic groups are segregated into the same areas, at the four spatial scales. This involves standardizing the covariance for each set of differences for each ethnicity at the relevant scale. The resultant correlation coefficients are reported in Table 6. Only those exceeding ±0.4 are shown, with positive coefficients in italics and negative coefficients in bold. The first block shows the correlations at the Borough (below the diagonal) and MSOA (above the diagonal) scales; the lower block does the same, respectively, for the LSOA and OA scales. One clear conclusion stands out: the sparseness of the matrices (the relatively small number of coefficients >±0.4) indicates that most distributions are relatively independent of each other, with few (especially strong) common patterns. This is particularly the case at the smaller scales: only 21 of the 78 coefficients are above that threshold in the Borough analyses; 25 at the MSOA scale; 13 at the LSOA scale; and just three at the OA. A finding of few positive correlations indicates that at all four scales, each of the groups has a distinct residential distribution, separate from that of most if not all of the 12 others.

**Table 6:** Cross-correlations comparing the pairwise distributions of the 13 groups at the four spatial scales

	WB	WI	WO	WBC	WBA	WA	I	P	Ba	C	BA	BC	A
Borough/MSOA													
White British	1.0	---	––	––	––	––	**–0.56**	**–0.68**	**–0.62**	––	**–0.51**	**–0.44**	**–0.41**
White Irish	––	1.0	––	––	––	*0.47*	––	––	––	––	––	––	––
White Other	––	*0.48*	1.0	––	––	*0.47*	––	––	––	*0.58*		––	0.50
White–BC	**–0.42**	––	*0.43*	1.0	*0.80*	*––*	*––*	*––*	*0.41*	––	*0.72*	*0.81*	––
White–BA	––	––	––	*0.57*	1.0	––	––	––	*0.48*	––	*0.83*	*0.76*	*0.53*
White–Asian	––	*0.53*	*0.59*	––	––	1.0	––	––	––	––	––	––	––
Indian	––	––	––	––	––	––	1.0	*0.71*	––	––	––	––	––
Pakistani	**–0.45**	––	––	––	––	––	*0.51*	1.0	*0.57*	––	––	––	––
Bangladeshi	––	––	––	––	––	––	––	––	1.0	––	*0.65*	*0.62*	*0.47*
Chinese	––	––	*0.44*	*––*	*––*	*0.42*	––	––	––	1.0	––	––	––
Black African	**–0.40**	––	––	*0.57*	*0.64*	––	––	––	––	––	1.0	*0.82*	*0.53*
BCaribbean	**–0.44**	––	––	*0.68*	*0.58*	*––*	*––*	*––*	*––*	––	*0.65*	1.0	*0.42*
Arab	**–0.42**	––	*0.55*	*––*	*––*	*0.53*	––	––	––	––	––	––	1.0
LSOA/OA													
White British	1.0	––	––	––	––	––	––	––	––	––	**–0.48**	––	––
White Irish	*0.47*	1.0	––	––	––	––	––	––	––	––	––	––	––
White Other	––	––	1.0	––	––	––	––	––	––	––	––	––	––
White–BC	––	––	––	1.0	––	––	––	––	––	––	––	*0.46*	––
White–BA	––	––	––	0.58	1.0	––	––	––	––	––	––	––	––
White–Asian	––	––	––	––	––	1.0	––	––	––	––	––	––	––
Indian	––	––	––	––	––	––	1.0	––	––	––	––	––	––
Pakistani	**–0.41**	––	––	––	––	––	*0.52*	1.0	––	––	––	––	––
Bangladeshi	**–0.44**	––	––	––	––	––	––	––	1.0	––	––	––	––
Chinese	––	––	*0.43*	*––*	*––*	*––*	*0.44*	––	––	1.0	––	––	––
Black African	**–0.43**	––	––	*0.68*	*0.66*	*––*	*––*	*––*	*0.58*	––	1.0	*0.42*	––
Black Caribbean	––	––	––	––	––	––	––	––	––	––	*0.78*	1.0	––
Arab	––	––	––	––	––	––	––	––	––	––	*0.40*	––	––

*Notes:* Positive coefficients are shown in italics, and negative coefficients are shown in bold. WB = White British; WI = White Irish; WO = White Other; WBC (White–BC = Mixed White–Black Caribbean; WBA (White–BA) = Mixed White–Black African; WA = Mixed White–Asian; I = Indian; P = Pakistani; Ba = Bangladeshi; C = Chinese; BA = Black African; BC = Black Caribbean; A = Arab.

The only substantial negative correlations in the four matrices apply to the White British population: at the Borough and MSOA scales, areas where there are many more White British residents than average tend to have fewer than average members of several of the Asian and Black ethnic groups. Among the positive correlations, some of the largest refer to the Black Africans and Black Caribbeans, plus those claiming a mixed White–Black African/Black Caribbean identity; these four groups cluster together in above average proportions at all scales. The only other clear pattern of clustering together—notably at the Borough and MSOA scales—is of Indians and Pakistanis, many of whom can be found not only in the same (western) sector of London (Johnston et al. 2014) but also clustered in major segments of those boroughs.

## Conclusions

This article has introduced a new procedure for measuring ethnic residential segregation, using a formal modeling strategy rather than the descriptive indices characteristic of most studies of that phenomenon. Because it can accommodate multigroup populations, it is ideally suited for investigations of segregation in large cities, most of which are characterized by several separate ethnic groups. The modeling procedure also operates at a variety of spatial scales and thus can evaluate the degree of segregation at different resolution levels. Further, because it is based on a formal modeling procedure that takes into account natural variation in the distribution of small absolute counts, the segregation estimates—produced using Bayesian procedures—have associated credible intervals, which allow statements to be made regarding the substantive significance of differences between groups in the degree of segregation (at each spatial scale).

We have demonstrated the usefulness of this single overall model for analyzing the degree of residential segregation for multiple ethnicities at multiple scales; and we have done so using data for the London metropolitan area in 2011, a very large study of 13 ethnicities at four spatial scales involving estimating more than one-third million rates (the ratios of observed to expected numbers in each of the areas for each of the 13 ethnic groups) for more than 8 million people. This multilevel framework can be applied elsewhere; in U.S. cities, for example, a hierarchical structure such as that deployed by Fischer et al. (2004) could be constructed and extended to include block groups. Moreover, the multilevel framework has the capacity for further important extensions, such as examining changing ethnic residential distributions (Owen and Jones 2015). The stochastic nature of the counts is of particular importance for this type of application because apparent secular differences could be found due to chance fluctuations over time. It is also possible to analyze nonhierarchical structures, such as cross-classifications, where the contexts are not strictly nested but crossed (Duncan et al. 1998). Such models could be used to analyze simultaneously school segregation net of residential segregation and vice versa. It is also possible to have models in which the variance is structured by explanatory variables to investigate, for example, how the degree of ethnic segregation depends on the amount of deprivation.

The substantive results from this initial application of the modeling strategy to ethnic segregation in London in 2011 illustrate the importance of investigating scalar differences. A general pattern emerges for several of the ethnic groups analyzed: their greatest concentration is at the largest and smallest scales, clustered both into certain segments of the city (at the Borough scale) and, within those segments, into groups of small areas. But that generalization does not apply to all the ethnic groups, indicating that segregation patterns are multifaceted and that the use of single-number indices suggesting that some are more segregated than others fails to uncover the full detail of a complex set of overlapping maps.

## Footnotes

1 The data can be obtained from Table KS201EW on the relevant websites (e.g., https://www.nomisweb.co.uk/).

2 It is included within the City of Westminster, with which it is joined in a Parliamentary constituency.

3 These responsibilities, though, are reducing because of administrative centralization by successive UK governments and the creation in 1990 of a separate Greater London Authority, with an elected mayor and assembly, which has powers over transport, policing, economic development, and the fire service.

4 This information is available online (http://www.londontown.com/LondonStreets/).

5 We exclude the rapidly expanding, heterogeneous “black other” group.

6 *D* measures the share of either group in a two-group total population that must be moved, without replacement, across the set of spatial units to achieve zero—a measure of how far away the distribution is from evenness. *D* will be upwardly biased because it represents the sum of the absolute value of the differences in proportions in an area. Natural stochastic variation will lead to nonzero differences when the counts are numerically small; and because the count cannot be negative, the index will be upwardly biased. Moreover, *D* is typically applied without any calculation of uncertainty, such as confidence intervals.

7 See Owen and Jones (2015) and Jones et al. (2014) for more details on this specification.

8 MCMC procedures (see later) estimate the overall intercept such that the population average value—the log rate in the mean area—is approximately zero. The cluster-specific value, which represents the log rate in the median area, can be much lower than this (Jones and Subramanian 2014b). The MLwiN software estimates the cluster specific estimates, but the customized predictions facility (Rasbash et al. 2012) allows the derivation of the population average values through simulation. Because the data and models are specified such that these log population average values are zero, we discuss them no further: the variance summarizes these differences from this even distribution.

9 The formula is $MRR= exp\left[\sqrt{2\times Variance}\times 0.6745\right]$; the value 0.6745 is the 75th percentile of the cumulative distribution function of the normal distribution with mean 0 and variance 1. The credible intervals for a MRR can be obtained by plugging in the credible intervals of the variance on the log scale obtained from a MCMC run.

10 Larsen and Merlo (2005) developed this approach originally for multilevel logit models (the median odds ratio), and Larsen (2006) later extended it to log Poisson models (naming it the *median mean ratio*). Consistent with Chan et al. (2011), we prefer the term *MRR* because it is a better descriptor of what it measures. The term *mean ratio* comes from the use of the Poisson model in the analysis of mean incidence rates, and Larsen was aiming to develop a comparable measure for the interpretation of random effects.

11 The estimated log differences from the empirical Bayes (EB) quasi-likelihood procedures were found in some cases (e.g., for the highly concentrated Bangladeshis) to be skewed, but this was reduced substantially when the full Bayes (FB) MCMC procedures were used.

12 In London, the OA with a median radius of 100m is considerably finer than their 500m pedestrian-based neighborhood; the borough scale with a mean radius of 3,922m is highly comparable to their macro-local environment.

13 For example, Dong and Harris (2015) and Dong et al. (2015) developed a multilevel model that not only has spatial dependence between higher-level areal units but also has additional dependency between lower-level units; Lee et al. (2014) used random effects to achieve subregions of localized smoothness with additional cluster fixed effects to model step changes between neighboring spatial units to guard against over smoothing across boundaries.

14 The matter in practice is more complicated due to the Level 1 Poisson nature of the σ_*e*1_^2^ variance; the full specification is given by Stryhn et al. (2006), who derived the intraclass correlation for Poisson responses in a multilevel model.

15 There is, of course, no implicit assumption of dependence at the MSOA level, but there would be if the higher borough level were included.

16 The prior distributions are specified as follows for the case of two ethnicities in a two-level model:

$$ \begin{array}{l}p\left({\upbeta}_1\right)\propto 1;p\left({\upbeta}_2\right)\propto 1\hfill \\ {}p\left(\left[\begin{array}{cc}\hfill {\upsigma}_{u1}^2\hfill & \hfill \hfill \\ {}\hfill {o}_{u12}\hfill & \hfill {\upsigma}_{u2}^2\hfill \end{array}\right]\right)\sim Inverse\kern0.75em  Wishar{t}_2\left(2\times \left[\begin{array}{cc}\hfill \widehat{\upsigma_{u1}^2}\hfill & \hfill \hfill \\ {}\hfill \widehat{o_{u12}}\hfill & \hfill \widehat{\upsigma_{u2}^2}\hfill \end{array}\right],2\right)\hfill \end{array} $$

The probability prior for the βs is given as a uniform distribution in which any value is equally likely. And for the between-OA variance–covariance matrix, we use a conjugate inverse Wishart prior distribution. It is based on the EB initial quasi-likelihood estimates in such a way as to be only mildly informative (Browne 2012:16). Browne and Draper (2000) examined the performance of this inverse Wishart prior distribution and chose it as the default in MLwiN. Gelman (2006) found that the choice of priors for the variance terms is of the greatest importance when there are few higher-level units and the variance is likely to be small. This does not apply here where, in the worst case, there are more than 30 highest-level spatial units (the Boroughs) and quite large variances are anticipated. Indeed, we also tried a uniform variance for the prior, but it made little difference.

17 These MRR values (and the variance estimates on which they are based) are relative and not absolute measures of segregation. We are currently exploring ways of transforming them into absolute measures.

18 This can be checked using the CIs reported in Tables 2 and 3.

19 That internal heterogeneity cannot be decomposed using the available census data on self-assessed ethnicity.

## References

[CR1] Allen, R., Burgess, S., Davidson, R., & Windmeijer, F. (2015). More reliable inference for the dissimilarity index of segregation. *Econometrics Journal, 18,* 40–66. 10.1111/ectj.12039 PMC5054828 27774035

[CR2] Bell A, Jones K (2015). Explaining fixed effects: Random effect modelling of time series, cross-sectional and panel data. Political Science and Research Methods.

[CR3] Breslow NE, Day NE (1987). Statistical methods in cancer research, volume II: The design and analysis of cohort studies.

[CR4] Browne, W. J. (2012). *MCMC estimation in MLwiN, v2.25*. Bristol, UK: University of Bristol, Centre for Multilevel Modelling. Retrieved from http://www.bristol.ac.uk/cmm/software/mlwin/download/manuals.html

[CR5] Browne WJ, Draper D (2000). Implementation and performance issues in the Bayesian fitting of multilevel models. Computational Statistics.

[CR6] Browne, W. J., Subramanian, S. V., Jones, K., & Goldstein, H. (2005). Variance partitioning in multilevel logistic models that exhibit over-dispersion. *Journal of the Royal Statistical Society: Series A, 168,* 599–614.

[CR7] Carrington WJ, Troske KR (1997). On measuring segregation in samples with small units. Journal of Business and Economic Statistics.

[CR8] Chan PS, Maddox TM, Tang F, Spinler S, Spertus JA (2011). Practice-level variation in warfarin use among outpatients with atrial fibrillation. American Journal of Cardiology.

[CR9] Cockings S, Harfoot A, Martin D, Hornby D (2011). Maintaining existing zoning systems using automated zone design techniques: Methods for creating the 2011 Census output geographies for England and Wales. Environment and Planning A.

[CR10] Cohen J (1988). Statistical power analysis for the behavioural sciences.

[CR11] Cummings G (2014). The new statistics: Why and how. Psychological Science.

[CR12] Dong G, Harris RJ (2015). Spatial autoregressive models for geographically hierarchical data structures. Geographical Analysis.

[CR13] Dong G, Harris RJ, Jones K, Yu J (2015). Multilevel modelling with spatial interaction effects with application to an emerging land market in Beijing, China. PloS One.

[CR14] Draper D, de Leeuw J, Meijer E (2008). Bayesian multilevel analysis and MCMC. Handbook of multilevel analysis.

[CR15] Duncan C, Jones K, Moon G (1998). Context, composition and heterogeneity: Using multilevel models in health research. Social Science & Medicine.

[CR16] Elffers H (2003). Analysing neighbourhood influence in criminology. Statistica Neerlandica.

[CR17] Fischer CS, Stockmayer G, Stiles J, Hout M (2004). Distinguishing the geographic levels and social dimensions of U.S. metropolitan segregation. Demography.

[CR18] Fotheringham AS, Brunsdon C, Charlton ME (2002). Geographically weighted regression: The analysis of spatially varying relationships.

[CR19] Fowler CS (2015). Segregation as a multiscalar phenomenon and its implications for neighbourhood-scale research: The case of South Seattle 1990–2010. Urban Geography.

[CR20] Gelman A (2006). Prior distributions for variance parameters in hierarchical models. Bayesian Analysis.

[CR21] Goldstein H (2011). Multilevel statistical models.

[CR22] Gorard S (2007). The dubious benefits of multi-level modelling. International Journal of Research and Method in Education.

[CR23] Jivraj S, Simpson L (2015). Ethnic identity and inequalities in Britain.

[CR24] Johnston RJ, Poulsen MF, Forrest J (2013). Multiethnic residential areas in a multi-ethnic country? A decade of major change in England and Wales. Environment and Planning A.

[CR25] Johnston RJ, Poulsen MF, Forrest J (2014). London’s changing ethnic landscape, 2001–2011: A cartographic exploration. Local Population Studies.

[CR26] Johnston RJ, Poulsen MF, Forrest J (2015). Increasing diversity within increasing diversity: The changing ethnic composition of London’s neighbourhoods, 2001–2011. Population, Space and Place.

[CR27] Johnston RJ, Voas D, Poulsen MF (2003). Measuring spatial concentration: The use of threshold profiles. Environment and Planning B: Planning and Design.

[CR28] Jones TP, McEvoy D (1978). Race and space in cloud-cuckoo land. Area.

[CR29] Jones K, Owen D, Johnston R, Forrest J, Manley D (2014). Modelling the occupational assimilation of immigrants by ancestry, age group and generational differences in Australia: A random effects approach to a large table of counts. Quality and Quantity.

[CR30] Jones K, Subramanian SV (2014). Developing multilevel models for analysing contexuality, heterogeneity and change.

[CR31] Jones K, Subramanian SV (2014). Developing multilevel models for analysing contexuality, heterogeneity and change.

[CR32] Kish L (1954). Differentiation in metropolitan areas. American Sociological Review.

[CR33] Larsen, K. (2006). *New measures for understanding the multilevel logistic regression model*. Paper presented at the Bochum Workshop on “Statistische Methoden fur korrelierte Daten,” Bochum, Germany. Retrieved from http://www.biometrie.uni-heidelberg.de/statmeth-ag/veranstaltungen/bochum06/Vortrag_Larsen.pdf

[CR34] Larsen K, Merlo J (2005). Appropriate assessment of neighbourhood effects on individual health: Integrating random and fixed effects in multilevel logistic regression. American Journal of Epidemiology.

[CR35] Leckie G, Goldstein H (2015). A multilevel modelling approach to measuring changing patterns of ethnic composition and segregation among London secondary schools, 2001–2010. Journal of the Royal Statistical Society: Series A (Statistics in Society).

[CR36] Leckie G, Pillinger R, Jones K, Goldstein H (2012). Multilevel modelling of social segregation. Journal of Educational and Behavioral Statistics.

[CR37] Lee BA, Firebaugh G, Matthews SA, Reardon SF, Farrell CR, O’Sullivan D (2008). Beyond the census tract: Patterns and determinants of racial residential segregation at multiple scales. American Sociological Review.

[CR38] Lee, D., Minton, J., & Pryce, G. (2014). *Inference for segregation indices in the presence of spatial autocorrelation*. Paper presented at the Royal Statistical Society Annual Conference, Sheffield, UK.

[CR39] Logan, J. R., Zhang, W., & Chunyu, M. D. (2015). Emergent ghettos: Black neighborhoods in New York and Chicago, 1880–1940. *American Journal of Sociology, 120,* 1055–1094. 10.1086/680680 PMC4597788 26046225

[CR40] Manley D, Fischer MM, Nijkamp P (2014). Scale, aggregation, and the modifiable areal unit problem. Handbook of regional science.

[CR41] Manley D, Johnston RJ, Jones K, Owen D, Tammaru T, van Ham M, Marcinczak M, Musterd S (2015). Occupational segregation in London: A new framework for modelling segregation. Socio-economic segregation in European capital cities: East meets West (regions and cities).

[CR42] McCullagh P, Nelder JA (1989). Generalized linear models.

[CR43] McCulloch CE, Neuhaus JM (2011). Misspecifying the shape of a random effects distribution: Why getting it wrong may not matter. Statistical Science.

[CR44] Moellering H, Tobler W (1972). Geographical variances. Geographical Analysis.

[CR45] Östh J, Clark WAV, Malmberg S (2015). Measuring the scale of segregation using *k*-nearest neighbor aggregates. Geographical Analysis.

[CR46] Östh J, Malmberg B, Andersson EK, Lloyd CD, Shuttleworth IG, Wong DWS (2014). Analysing segregation using individualised neighbourhoods. Socio-spatial segregation: Concepts, processes and outcomes.

[CR47] Owen, D., & Jones, K. (2015). *Geographical inequalities in mortality: A model-based approach to analysing fine-grained differences over time: England and Wales, 2002–2012*. Unpublished manuscript, School of Geographical Sciences, University of Bristol, Bristol, UK.

[CR48] Paelinck J, Klaassen L (1979). Spatial econometrics.

[CR49] Rasbash J, Charlton C, Browne WJ, Healy M, Cameron B (2014). MLwiN version 2.31.

[CR50] Rasbash J, Charlton C, Jones K, Pillinger R (2012). Manual supplement to MLwiN v.2.26.

[CR51] Reardon SF, Farrell CR, Matthews SA, O’Sullivan D, Bischoff K, Firebaugh G (2009). Race and space in the 1990s: Changes in the geographic scale of racial residential segregation, 1990–2000. Social Science Research.

[CR52] Reardon SF, Firebaugh G (2002). Measures of multigroup segregation. Sociological Methodology.

[CR53] Reardon SF, Matthews SA, O’Sullivan D, Lee BA, Firebaugh G, Farrell CR, Bischoff K (2008). The geographical scale of metropolitan racial segregation. Demography.

[CR54] Reardon SF, O’Sullivan D (2004). Measures of spatial segregation. Sociological Methodology.

[CR55] Spiegelhalter DJ, Best NG, Carlin BP, van der Linde A (2002). Bayesian measures of model complexity and fit. Journal of the Royal Statistical Society: Series B.

[CR56] Stryhn H., Sanchez J., Morley P., Booker C., & Dohoo, I. R. (2006). Interpretation of variance parameters in multilevel Poisson regression models. *Proceedings of the 11th International Symposium on Veterinary Epidemiology and Economics*. Retrieved from www.sciquest.org.nz/T4-1.2.3+-+Interpretation+of+variance+parameters+i.pdf

[CR57] Sturgis P, Brunton-Smith I, Kuha J, Jackson J (2014). Ethnic diversity, segregation and the social cohesion of neighbourhoods in London. Ethnic & Racial Studies.

[CR58] Subramanian SV, Duncan C, Jones K (2001). Multilevel perspectives on modelling census data. Environment and Planning A.

[CR59] Tranmer M, Steel D (2001). Ignoring a level in a multilevel model: Evidence from UK census data. Environment and Planning A.

[CR60] Voas D, Williamson P (2000). The scale of dissimilarity: Concepts, measurement and an application to socio-economic variation across England and Wales. Transactions of the Institute of British Geographers.

[CR61] Wong DWS (1998). Measuring multi-ethnic spatial segregation. Urban Geography.

[CR62] Wong DWS (2003). Spatial decomposition of segregation indices: A framework toward measuring segregation at multiple levels. Geographical Analysis.

[CR63] Woods RI (1976). Aspects of the scale problem in the calculation of segregation indices: London and Birmingham, 1961 and 1971. Tijdschrift voor Economische en Sociale Geografie.

[CR64] Wright, R., Ellis, M., Holloway, S., & Wong, S. (2011). Patterns of racial segregation and diversity in the United States: 1990–2010. *Professional Geographer, 66,* 173–182. 10.1080/00330124.2012.735924 PMC4114976 25083001

